# Conflict resolution in the case of convective weather cell circumvention

**DOI:** 10.1186/s40537-023-00759-8

**Published:** 2023-05-25

**Authors:** Vojislav Bogdanovic, Zhi-Hong Mao

**Affiliations:** Department for Air Transport and Traffic, Faculty of Transport and Traffic Engineering, University of Belgrade, Vojvode Stepe 305, 11000 Belgrade, Serbia

**Keywords:** Air traffic control (ATC), Conflict resolution, Intersecting flows, Convective weather cell, Unmanned aerial vehicles (UAV), Big Data

## Abstract

This research analyzes the area required for the conflict resolution between aircraft in two flows impacted by a convective weather cell (CWC). The CWC is introduced as a constrained area, forbidden to flight through, which affects the air traffic flows. Prior the conflict resolution, two flows and their intersection are relocated away from the CWC area (thus enabling circumvention of the CWC), which is followed by a tuning of the relocated flows intersection angle in order to create the minimal size of the conflict zone (CZ—a circular area centered at the intersection of two flows, which provides aircraft enough space to completely resolve the conflict within). Therefore, the essence of the proposed solution is in providing conflict free trajectories for the aircraft in intersecting flows that are affected by the CWC, with the goal of minimizing the CZ size, so the finite occupied airspace for the conflict resolution and the CWC circumvention could be reduced. Compared to the best solutions and current industry practice, this article is focused in reduction of the airspace required for aircraft to aircraft and aircraft to weather conflict resolution, and not to distance travelled, time savings, and fuel consumption minimization. The conducted analysis in the MicrosoftExcel2010 confirmed the relevance of the proposed model and demonstrated variations in efficiency of the utilized airspace. The proposed model's transdisciplinary nature makes it potentially applicable in other fields of study, such as the conflict resolution between unmanned aerial vehicles (UAVs) and fixed objects like buildings. Building on this model and taking in consideration large and complex data sets, such as weather related data and flight data (aircraft position, speed, and altitude), we believe it is possible to conduct more sophisticated analyses that would take advantage of Big Data.

## Introduction

Today’s air traffic control (ATC) is faced with challenges such as rapid demand growth, severe weather impact, and increased air traffic controllers (ATCo) workload, which stand in a way of further improvement of the ATC and therefore the air traffic.

Prior COVID-19 crisis (until 2019), global air traffic continued to follow the growth rate. From the analysis conducted by ICAO in 2015 (based on the sources: ICAO, IATA, OAG) [1], we may see that in 2015 world passenger traffic grew by + 6.8% on revenue passenger kilometer (RPK), which is + 1.0 percentage points higher than the growth in 2014 (+ 5.8%). Data in the same analysis conducted for air cargo showed that the world freight traffic (FTK) was increased by + 2.2% on year-by-year basis in the 2015, which is less than a half of the 2014 growth registered (+ 4.9%). In the following years ICAO reports showed: increase of + 7.9% in RPKs (total of 7699 billion RPKs) and + 9.5% in FTKs (total of 56 million tonnes of freight carried) compared to 2016 [2]; increase of + 7.1% in RPKs (total of 8258 billion RPKs) and + 3.6% in FTKs (total of 58.0 million tonnes of freight carried) compared to 2017 [3]; increase in + 4.9% in RPK (total of 8686 billion RPKs) and slight decrease of − 2.9% in FTKs (total of 57.6 million tonnes of freight carried) compared to 2018 [4].

During COVID-19 period global air traffic and transport faced significant decline. ICAO report for 2020 [5] showed − 65.5% decline in RPKs (total of 2990 billion RPKs) and − 16.7% decline in FTKs (total of 48.9 million of tonnes carried). For 2021 and 2022 ICAO has not published annual statistical reports. In its Annual Review 2021 IATA stated that RPKs remained down by − 64.5% between January 2021 and July 2021 compared to the same period in pre-crisis 2019. On the other side, in the first half of 2021 the air cargo marked + 7.9% increase in FTKs compared to the same period in 2019. It even surpassed pre-crisis peak from August 2018 by + 5% [6]. From IATA’s Annual Review 2022 air passenger traffic kept the trend of recovery, being on -37.2% in RPK compared to 2019. Air cargo traffic continued to grow in year-on-year terms, but the growth intensity was softened in the second half of 2021 and is on a decline roll [7]. From historical point of view, the air traffic bounced back after each crisis and marked even better results in the coming period (i.e., after 09.11. crisis and Global Financial Crisis). Therefore, expecting air traffic peak results exceedance and further growth in the upcoming period is fairly justified. Forecasts conducted by Airbus, for the long-term period from 2016 to 2035, show that the passenger air traffic will grow annually by 4.5% in the next 20 years [8]. All these data indicate that the ATC must be prepared to provide capacity as a sufficient response for the forecasted demand.

Providing capacity for the forecasted demand will not resolve all the problems. Related to it, another major challenge for the ATC presents severe weather (e.g., Hurricanes, Storms, Cumulonimbi), which may have a huge impact on the flow of the air traffic. The severe weather appearance may result in decrease of available capacity of an airspace sector, and in some cases it can lead to closure of parts of airspace sectors or the whole sector, which is often followed by an increased air traffic in vicinity sectors. These situations could lead to imbalance between capacity and demand of the vicinity sectors where the demand may exceed the capacity. In such cases, the air traffic becomes more complex, and hence affects the ATCo workload and the overall air traffic safety. As more aircraft are feeding the sectors in vicinity, they are creating more potential conflicts that must be resolved without causing new ones (the domino effect). A conflict is declared whenever separation distance between two aircraft is less than the required minimum. Therefore, the ATCo must constantly observe separation between aircraft, aircraft and weather cells, and look out for separation distances that could be below the declared minimum. In such situations, with high intensity of air traffic, working environment of the ATCo becomes aggravated, requiring more awareness and resulting in an increased ATCo workload.

In most cases severe weather appearance entails delays also. This is due to detour around an affected area, as well as applied holding procedures in order to make the aircraft wait for better weather conditions. The severe weather can also result in cancelled flights, but this is usually due to closed airports.

Out of all delays occurred in air traffic, 70% of them are caused by the adverse weather (synonym for the “severe weather”), where 60% of weather related delays is due to convective weather [9]. As the convective weather has major contribution in weather-related delays, here we will consider mitigating its impact by providing a solution for its avoidance.

The goal of the research, besides providing efficient CWC avoidance, was to minimize the area required for a conflict resolution, and to configure that area with the CWC area, so the finite occupied airspace could also be minimized.

The research article is organized as follows: The section "Problem statement" addresses the problem statement and reviews the assumptions and definitions of conflict resolution in accordance to previous research of Mao et al. The sections "Existing solution" and "Developed algorithms for the conflict resolution between aircraft and the CWC" cover significant existing solutions related to the research problem. The section "Proposed solution" introduces assumptions and conditions of the proposed solution and developed model. The analysis and results are presented in the section titled "Analysis of the solution—a numerical example". The section "Extension to other field of study" explores the potential of the developed model for application in other fields of study, such as conflict resolution between unmanned aerial vehicles and fixed objects. The conclusion is the final section of the research paper.

## Problem statement

The CWC avoidance is accomplished through flying via trajectories that lead aircraft around convective weather. While flying via these trajectories, aircraft needs to avoid potential conflicts with other aircraft with whom the trajectories are intersecting. Areas around intersection points between flows (IPts) need to be large enough, so that all potential conflicts can be resolved within those areas, but without causing penetration through the CWC. Therefore, from the safety aspect it is important to obtain conflict-free trajectories to all observed aircraft, and at the same time to maintain at least the minimum distance required between aircraft and the CWC. Besides safety, it is desirable to route the aircraft via shortest possible paths in order to reduce flight time and achieve fuel efficiency. Also, from the ATCo point of view, benefits due to such efficient air traffic flow could be a reduced workload and a more available airspace (significant from the capacity-demand aspect) in already constrained and unfavorable conditions. Considering forecasts and hence anticipated significant air traffic growth, the problem will certainly become more serious over time.

**Fig. 1:**
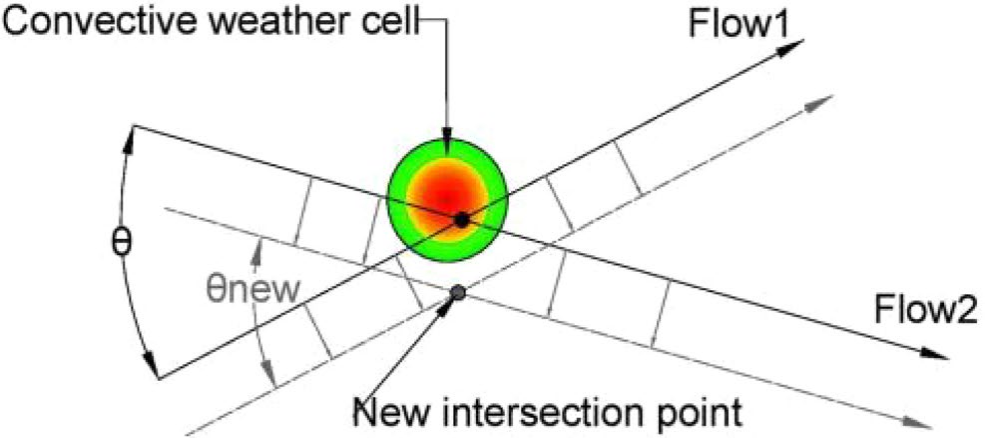
Example of CWC avoidance by the flows displacement. Original flows—black lines (intersection angle θ); displaced flows—grey dashed lines (intersection angle ${{\varvec{\theta}}}_{{\varvec{n}}{\varvec{e}}{\varvec{w}}}$). Intensity of the CWC impact: red—highest intensity; green—lowest intensity

In a simple case, with only two intersecting flows, the CWC avoidance could be obtained by displacing the original flows, as well as the IPt, in a way as it was illustrated in Fig. 1. Depicted CWC already accounts the minimum separation distance between the aircraft and the CWC of 20 NM, proposed by the FAA [10].

Relocation of the IPt will be constrained by the required area for the conflict resolution between aircraft in two flows, known as the *conflict zone* (CZ), where the CZ must be located on a safe distance from the CWC (in order to prevent potential penetrations). In [11, 12] the CZ is defined as a circular area centered at the IPt of two flows, which provides aircraft enough space to completely resolve the conflict within the area, by performing required conflict resolution maneuvers (CRM).

**Fig. 2:**
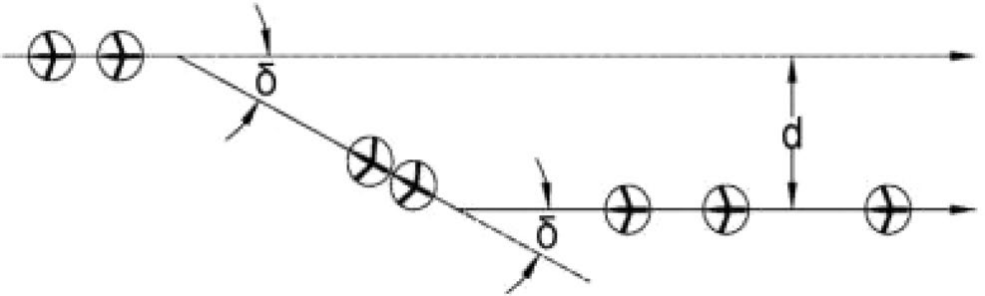
Aircraft lateral displacement realized by a two-heading-change maneuver

As per [13], aircraft entering the CZ perform maneuvers one at a time while accounting for all other aircraft that have already completed CRM as moving obstacles. Aircraft outside the CZ and those that have not yet maneuvered are not considered. While within the CZ, all necessary maneuvers must be completed, and before leaving the CZ, aircraft must return to their original tracks. The CRM maneuvers carried out within the CZ ensure that aircraft have a conflict-free trajectory. As explained in [6], the CRM consists of the offset inception maneuver (which aircraft initiates at the entry point of the CZ, maintains desired lateral displacement, and completes it before crossing the other flow) and the offset return maneuver (which aircraft initiates after crossing the other flow, returns back to the original heading, and completes it within the CZ). Abovementioned lateral displacement, illustrated in Fig. 2, provides conflict avoidance between entering aircraft and any other aircraft within the CZ, and its amplitude is as small as possible.

As the FAA’s lateral separation distance between aircraft is 5 NM (${\mathrm{D}}_{\mathrm{SEP}}$ = 5 NM) for en route traffic, we can consider an aircraft as circle of radius r = 2.5 NM (which is ${\mathrm{D}}_{\mathrm{SEP}}/2$). Thus, the considered circle around an aircraft presents the aircraft protected zone. So, from the conflict resolution aspect, it is strictly important that protected zones between aircraft do not overlap; otherwise, the conflict will occur. From the Fig. 2, for heading change magnitude (δ) and lateral displacement d, in order to complete a maneuver, aircraft will need to travel a distance ${}^{d}/{}_{\sin \delta }$, while the longitudinal distance in travel will be ${}^{d}/{}_{\tan \delta }$.

As per [14], the magnitude of the lateral displacement required for the conflict resolution is bounded above by (1), where the intersection angle between two flows is on interval θ ϵ (0,π):

1 $${d}_{max}=\frac{{D}_{sep}}{\mathrm{sin}\frac{\theta }{2}}$$

**Fig. 3:**
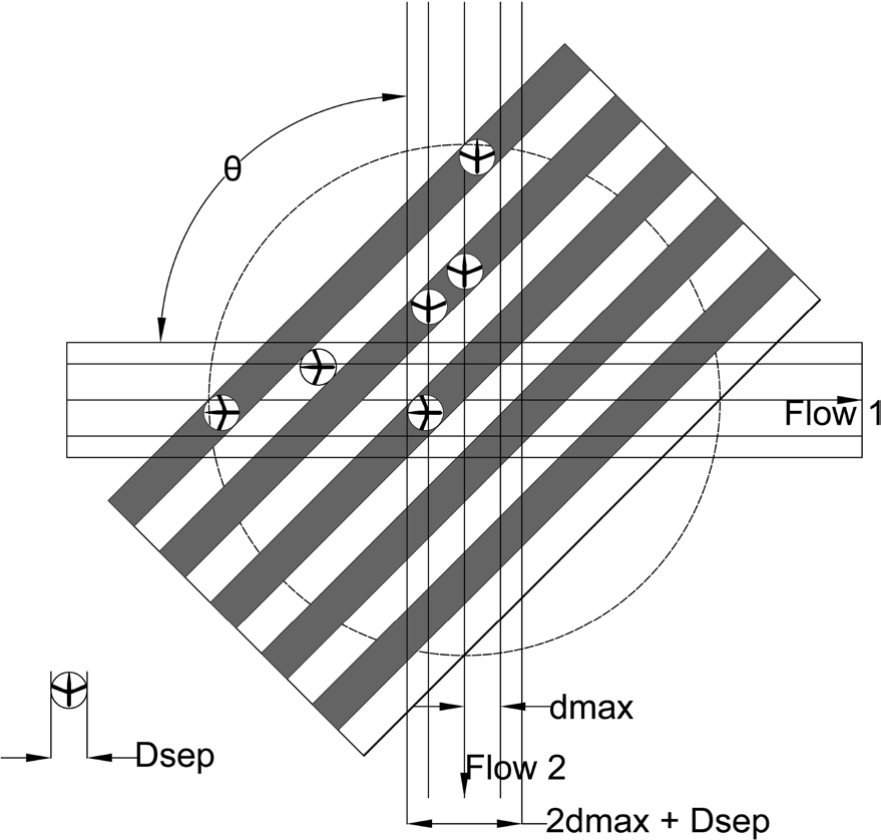
The conflict resolution for two intersecting flows using lateral displacement

Conflict resolution for the intersection of two flows, using lateral displacement, can be understood in terms of aisles, as explained in [11, 12, 15]. We can project a linear slab-shape aisle of the width equal to ${D}_{SEP}$, centered at each aircraft and parallel to the relative velocity vector between flows ${F}_{1}$ and ${F}_{2}$, as illustrated in Fig. 3. Aircraft in each aisle move together with the aisles (there is no relative movement between the aircraft and the aisle in direction perpendicular to the aisles, but along the direction of aisles relative movement exists). Aisles of the flows ${F}_{1}$ and ${F}_{2}$ do not overlap, and the protected zones of aircraft within the aisles do not overlap neither (because of the aisles width ${D}_{SEP}$). So, if each aircraft inside its flow follows this concept, the conflict free trajectories will be obtained for all aircraft.

Now that we understand the maneuvers that aircraft need to perform within the CZ, and the principle of the conflict resolution within it, we can delve into how the CZ is calculated. The size of the conflict zone was studied by Mao et al. in [11, 12], where they derived formulas (2)–(5) to determine the CZ radius (r):

2 $$r={R}_{IN}+B \cdot {d}_{max}$$

3 $${R}_{IN}=\mathrm{max}\left\{{r}_{1},{r}_{2}\right\}+\frac{{D}_{sep}}{2}$$

4 $${r}_{1}=\frac{{D}_{sep}}{{\left(\mathrm{sin}\frac{\theta }{2}\right)}^{2}}+\frac{{D}_{sep}}{2\mathrm{sin}\frac{\theta }{2}};\quad {r}_{2}=\frac{{2D}_{sep}}{\mathrm{sin}\theta }+\frac{{D}_{sep}}{2\mathrm{cos}\frac{\theta }{2}}$$

5 $$\mathrm{max}\left\{{r}_{1},{r}_{2}\right\}=\left\{\begin{array}{c}\frac{{D}_{sep}}{{\left(\mathrm{sin}\frac{\theta }{2}\right)}^{2}}+\frac{{D}_{sep}}{2\mathrm{sin}\frac{\theta }{2}} \quad if 0<\theta \le \frac{\pi }{2}\\ \frac{{2D}_{sep}}{\mathrm{sin}\theta }+\frac{{D}_{sep}}{2\mathrm{cos}\frac{\theta }{2}} \quad \,\, if \frac{\pi }{2}<\theta <\pi \end{array}\right.$$

**Fig. 4:**
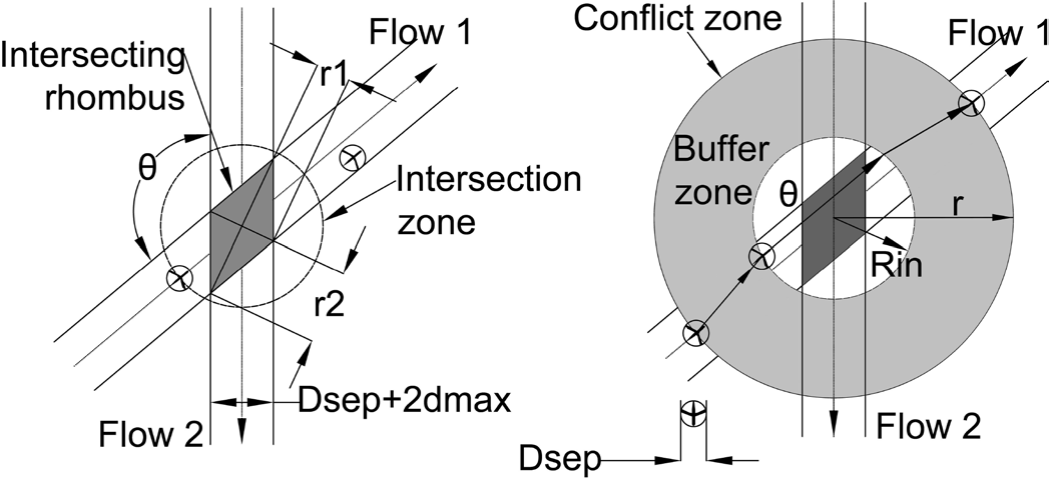
Intersection zone and intersecting rhombus diagonals (left). Intersecting rhombus, intersection zone, buffer zone, and CZ (right)

Value ${R}_{IN}$ is the radius of the intersection zone. It is determined using formulas (3) and (4), and it guarantees that the intersection zone fully covers the intersection rhombus (the rhombus is a consequence of the flows intersection). Values ${r}_{1}$ and ${r}_{2}$ are lengths of the two intersecting rhombus diagonals, while ${d}_{max}$ is the upper bound of the lateral displacement determined by (1). Value *B* is the buffer coefficient which provides an aircraft some lead space to determine required lateral displacement maneuver. As per [12], ${B\cdot d}_{max}$ in formula (2) presents width of the buffer zone. It ensures that aircraft will have enough space to complete the offset inception maneuver before entering the intersection zone, and also to complete the offset return maneuver after leaving the intersection zone (Fig. 4).

**Fig. 5:**
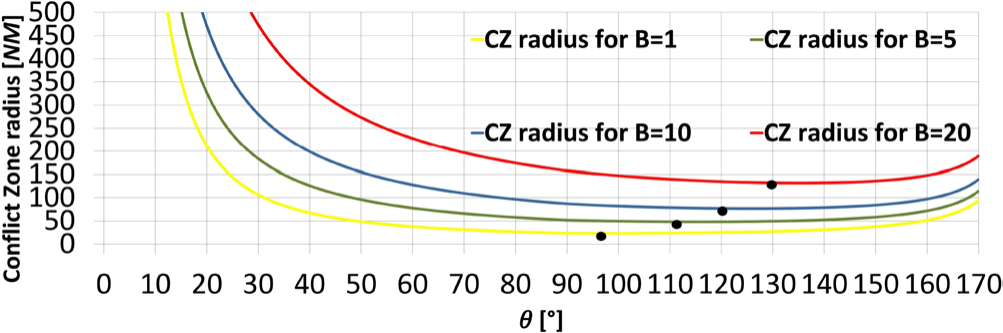
CZ radius (r) vs. the intersection angle (θ)

Huang et al. studied the optimal intersection angle (the angle for which the radius of the CZ (r) is minimal) in [12]. They discovered that the optimal intersection angle (${\theta }^{*}={\theta }_{opt}$), for different values of B, is on the interval ${\theta }^{*}$ ϵ $\left[\frac{\pi }{2},\pi \right.)$. In Fig. 5 the plot of r versus θ curves is illustrated for different values of B, and calculated using formulas (2)– (5). As buffer coefficient B increases, the ${\theta }^{*}$ will also increase, approaching 180°. Conversely, as B approaches to 0, ${\theta }^{*}$ will approach 90°.

It can be seen that the size of the CZ depends on θ for the fixed value of B. According to Huang et al. [12], the CZ concept may not be very efficient for small angles of intersection between flows. This can also be observed in Fig. 5. The reason for this insufficiency is due to fact that the CZ radius is a function of the intersection angle θ, and as θ approaches zero the CZ radius approaches infinity.

**Fig. 6:**
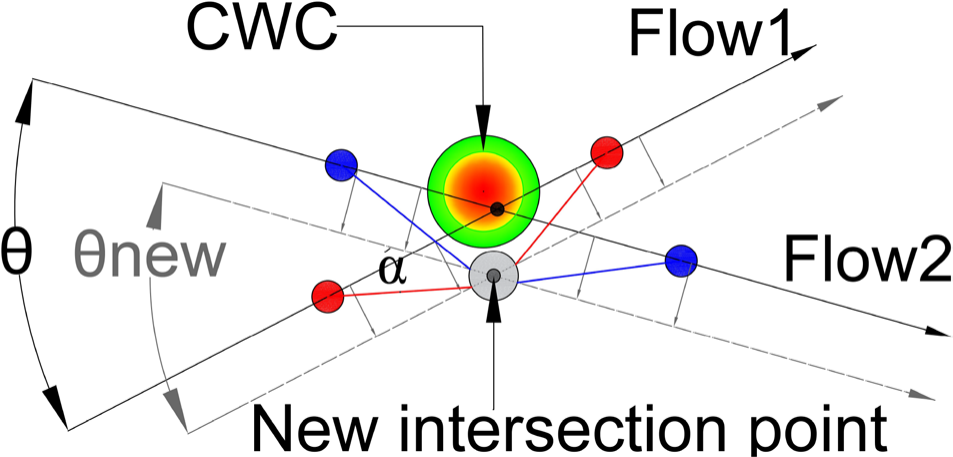
Potential conflict resolution in the case of CWC circumvention

When projecting new flows, it's important to examine angles around ${\theta }^{*}$ first. If ${\theta }^{*}$ cannot be applied, the chosen angle of intersection ${\theta }_{new}$ (angle of intersection between new-projected flows) should provide the smallest possible CZ and safe circumvention of the CWC. Applying the ${\theta }^{*}$ to the new-projected flows is particularly significant when the angles between the original flows and new-projected flows are similar, so that aircraft do not have to travel long distances to reach the CZ. However, if the original angle of intersection differs greatly from the new angle of intersection (especially when θ is small), the entry points of the CZ for both flows may be very distant from the original flows, resulting in significant deviations from the original heading (denoted as α in Fig. 6) to reach the entry point. While the ${\theta }^{*}$ configuration may reduce the size of the CZ, it may not be the best solution in terms of flight time and fuel consumption. Potential solution would be more significant if during the conflict resolution maneuvers aircraft also circumvent the CWC. In such case, after coming to the CZ exit point, aircraft will successfully resolve conflicts with other aircraft and the CWC, as illustrated in Fig. 6.

## Existing solution

**Fig. 7:**
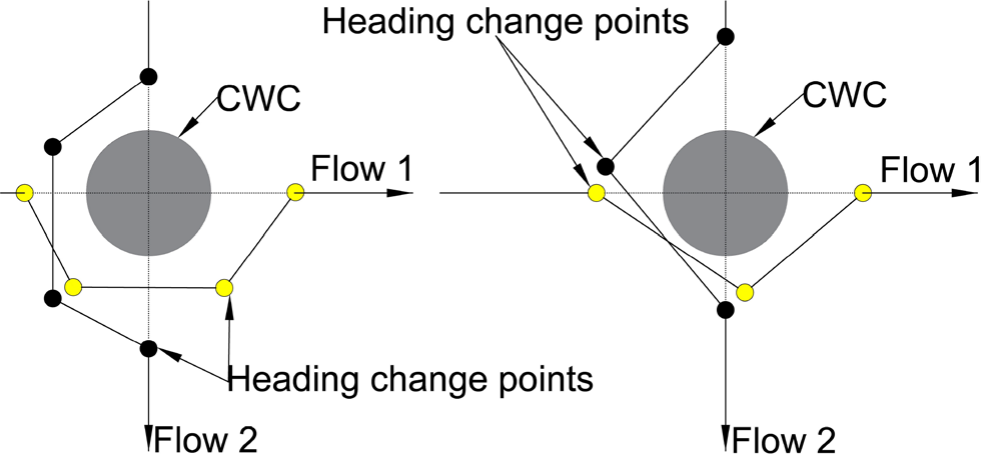
ATCo vectoring aircraft around the CWC—consequent trajectories

The current operational solution for avoiding CWC is to vector the aircraft around it by the ATCo. The ATCo issue a series of vectors for pilots, which consist of heading changes followed by a straight flight of certain duration (e.g., 1 or 2 min) or distance. This method is effective when the airspace is not congested, and pilots can easily follow the instructions, which facilitates their workload. The two examples of this solution are illustrated on Fig. 7, with the number of heading change points being the only difference between the two scenarios. In both figures, yellow circles represent aircraft in flow 1, while black circles denote aircraft in flow 2. The paths for circumventing the CWC consist of trajectories between the heading change points, after which all aircraft return to their original heading.

## Developed algorithms for the conflict resolution between aircraft and the CWC

Several algorithms for resolving conflicts between aircraft and a CWC have already been developed. Depending on the optimization objective, algorithms provide sufficient solutions in term of distance travelled or fuel and time savings. In this section we will briefly describe the most significant solutions.

### Extension of the algorithm for aircraft to aircraft conflicts in the Center-TRACON automation system

Love et al. extended an algorithm developed by Erzberger for aircraft to aircraft conflict avoidance in the Center-TRACON Automation System in [16]. In [17] Erzberger developed the algorithm for aircraft to aircraft conflict resolution, which can handle all combination of conflict types (between descending, climbing, and cruising aircraft) that may occur in en-route airspace.

In order to define weather constraints and regions, Love et al. used CWAM (Convective Weather Avoidance Model) integrated in the Center-TRACON Automation System.

The simulation results of the algorithm demonstrated a high success rate in resolving conflicts, both aircraft-to-aircraft and aircraft-to-weather. Specifically, the algorithm was able to resolve 96% of aircraft-to-aircraft conflicts without any weather constraints, while achieving a resolution rate of 91% for conflicts occurring in high traffic scenarios with moderate weather. In scenarios with bad weather and high traffic, the algorithm resolved 72% of the conflicts. These results highlight the effectiveness of the proposed algorithm in managing conflicts in various weather and traffic conditions.

Depending on the number of other aircraft in the vicinity, the location and size of individual weather polygons, and total area coverage, the success rate may vary. Also, comparing to the no weather scenario, the total number of conflicts for the bad weather scenario, for the medium and high traffic scenarios, was six times and four times bigger.

### Integrated algorithms for loss-of separation conflicts, sequencing and merging of arrival traffic, and weather cell avoidance

In [18] Erzberger et al. developed integrated algorithms for resolving three types of problems such as: separation conflicts, arrival sequencing, and weather-cell avoidance. The algorithms for separation conflicts and weather-cell avoidance are of the significance to be mentioned here and therefore we will describe their integration. The algorithm for weather-cell avoidance is designed to resolve conflicts with a weather-cell that is relatively close to the current aircraft position (from 4 to 30 min in time range) and considers only horizontal maneuvers. A weather-cell is considered to be of irregular shape, while several cells with a narrow corridor between them are assumed to be merged in a large cell.

For the weather-cell area specification, a bit-map is used. Prior the algorithm calculation, a polygon is projected to the boundary of the weather-cell, so the algorithm calculates trajectories around the polygon.

The algorithm starts computing the ray tangent to the weather-cell polygon, which emanates from current aircraft position. Second ray is calculated from the specified return-point (the point of return to original heading after weather-cell circumvention), that is also tangent to the weather-cell. First and second ray are intersecting at a point that is called the auxiliary-waypoint. With projection of two rays, the path around weather-cell is obtained. Also, the algorithm calculates the path in the opposite turn direction (from other side of the weather-cell), compares it with the first obtained path, and chooses the shorter of those two. Further, the coordinates of auxiliary-waypoint and return-waypoint are sent to Trial Conflict Probe and Trial Trajectory Engine, to check for potential conflicts and generate an avoidance trajectory. For induced conflicts, the Resolution-Generator finds conflict resolution considering constraints by preference order and suitable resolution types. Resolution that includes change of altitude is preferred as it preserves weather-avoidance path, and in the case it is unsuccessful, a path-stretch maneuver is considered, where the auxiliary waypoint is chosen to be a return waypoint for the path stretch maneuver.

Trajectories determined by this procedure (with one auxiliary-waypoint) are suitable for heading changes less than 90°. For greater angle changes, the procedure includes two auxiliary-waypoints. Calculation of the resolution trajectory with two auxiliary-waypoints starts the same as the calculation for the resolution with one auxiliary-waypoint. Two rays tangent to the weather-cell polygon are projected. For the first auxiliary-waypoint the trial location is chosen, which is on a short distance from the tangency point of the first tangent ray, then a third ray that emanates from the first auxiliary-waypoint (also a ray tangent to the weather-cell polygon) is projected. At the point of intersection between the second and the third ray, the second auxiliary-waypoint is located. If it happens that the location of the first auxiliary-waypoint does not provide a tangency line with the second projected ray, than the location of the first auxiliary-waypoint is moved in increments until this condition is satisfied.

This research gives contribution in providing the shortest possible path around weather-cell, and in cases of potential conflicts with other aircraft, the weather avoidance algorithm is in conjunction with the algorithm for loss-of separation conflicts, so the conflict-free trajectories could be obtained without penetrating the weather-cell.

This research was extended in [19], where Erzberger et al. presented design approach for basic algorithms for a future system, with a high level of autonomy, that can resolve aircraft conflict resolution, perform arrival scheduling and provide convective weather avoidance in terminal area airspace. Severity and location of a convective weather cells are detected by weather sensors, then processed by embedded convective weather analysis software. The software assigns risk levels for regions of airspace around the convection cells, and based on the input algorithms in [20] generate enclosing polygons. Afterwards, Terminal Auto Resolver proposed in [21], uses these polygons as inputs for generating conflict-free avoidance trajectories.

### A weather avoidance system for near-term trajectory-based operations (dynamic weather routes)

In [22] McNally et al. developed a ground-based trajectory automation system that continuously analyses flights in en-route airspace and calculates the time and fuel corrections to the already existing weather avoidance routes.

The system proposes reroutes—Dynamic Weather Routes (DWR) every 12 s, and provides visualization and modification of the routes, flying savings (fuel and time) evaluation, proximity to weather, traffic congestion, and traffic conflicts. The DWR system integrates the Center TRACON Automation System (CTAS) (configured for en-route Center operations), Convective Weather Avoidance Model (CWAM), and Future ATM Concepts Evaluation Tool (FACET).

McNally et al. conducted 14 h of analysis of the Fort Worth Center traffic over five convective weather days and they found the DWR routes for 171 flights with an average savings of about 10 min per flight. Also, traffic congestion decrease for a downstream sector (home Center (Fort Worth) and its immediate neighboring Centers: Kansas City, Memphis, Houston, Albuquerque) is notable if all aircraft are to fly via DWR routes.

Besides abovementioned articles, it is important to note a recent research conducted by Zhao et al. in [23], where a new multiple-aircraft-conflict resolution method was proposed that could be extended to tackle the problem of convective weather cell avoidance as well. The method is based on a probabilistic conflict risk map (image) that makes the conflict resolution problem equivalent to the problem of finding a path to avoid risks. As stated in the article, the conflict risk map calculation could be easily extended to take in consideration the other safety threats such as severe weather condition and restricted airspace. The risk avoiding paths are found using A* algorithm (finding the cost-minimized trajectory for a single aircraft by considering all other aircraft as intruders), while trajectory planning optimization is achieved by implementing search heuristic method for iterating the A* algorithm for all aircraft.

Depending on the point of view, mentioned solutions in this section give huge contribution to the aircraft to aircraft and/or the aircraft to weather conflict resolution. Algorithms are successful in resolving conflicts and providing the conflict free trajectories around the convective weather polygons. The conflict free trajectories are sufficient from the aspect of distance travelled, time savings, and fuel consumption, but these algorithms do not take into consideration the area reserved for the conflict resolution and its minimization, which may be significant in dense sectors during severe weather appearance.

In the following chapter, we present our proposed solution, which takes into account the intersection of flows and aims to minimize the area allocated for conflict resolution.

## Proposed solution

As previously discussed in the Problem Statement section (case illustrated in Fig. 1), our proposed solution takes into account two intersecting flows (${F}_{1}$ and ${F}_{2}$) that intersect at an angle θ, and the presence of the CWC located near the IPt which disrupts the air traffic flow. This necessitates the relocation of both the flows and the intersecting point.

We considered following conditions and assumptions:

1. The CWC is circular shaped with the radius ${\mathrm{R}}_{\mathrm{S}}$ that already accounts the minimum safety buffer of 20 NM;
2. The CWC is static (there is no movement of the cell);
3. The angle θ between flows ${\mathrm{F}}_{1}$ and ${\mathrm{F}}_{2}$ is arbitrary on interval θ ϵ (0,180);
4. We consider flows as single lines (single lane flows);
5. CRMs are in planar space only (all maneuvers are horizontal and all aircraft are flying at the same altitude);
6. There is no wind effect.

The first two assumptions do not cover real-world scenarios, in which the CWC’s shape is irregular and variable, while the cells are continuously moving. Consequently, treating the cell as a circular shape is only applicable if the projected circle encompasses the entire static cell. Even then, the optimal solution for which the CZ size is minimal, might not be the best solution possible due to the unutilized airspace left behind as a result of approximating the cell's shape with circle. Therefore, this research lays the groundwork for comprehending the problem and provides guidance for future investigations.

Following the assumptions, our goal is to find the smallest possible area of the CZ near-by the CWC, so the utilization of the occupied airspace by the aircraft in circumvention can be increased. Therefore, reducing the size of the CZ zone will result in reducing the area of airspace occupancy, and consequently, in increasing the available airspace for other aircraft.

The proposed solution would be more significant if paths in circumvention are as short as possible, thus the aircraft will sooner leave the affected airspace and reduce delays and fuel consumption (this will also help in preventing potential congestions). However, distances in circumvention depend on difference between the angle θ and the angle ${\theta }_{new}$. When the difference between these angles is larger, the deviations from the original and new-projected flows will also be larger. As a result, circumvention paths will be longer. Therefore, the paths in circumvention consist of:

1. Distance between current aircraft position and CZ entry point;
2. Distance in maneuvering within the CZ;
3. Distance between the CZ exit point and the point of return to original heading after the CWC circumvention.

It is important to note that we have been focused on calculating and minimizing the size of the CZ, so the calculation of distances and time in circumvention or fuel consumption are not covered in this research.

### Projection of the lines tangent to the CWC

**Fig. 8:**
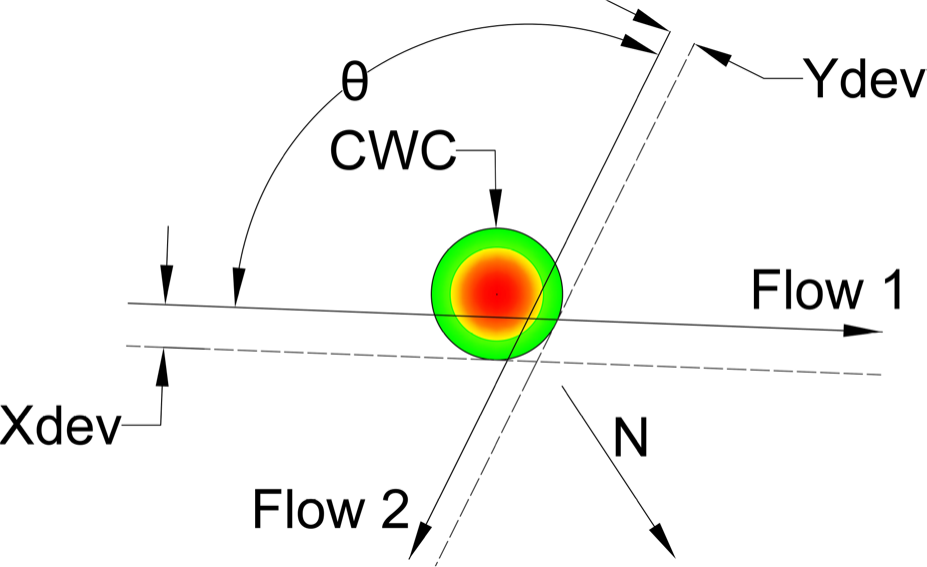
CWC, original and new-projected flows (dashed lines)

In Fig. 8, the disruption of air traffic flows caused by the CWC is illustrated for an obtuse angle of intersection.

To minimize the impact of the CWC on air traffic flows, the circumvention will be carried out from the side where the CWC has the least effect. Specifically, for aircraft in flow 1 (as shown in Fig. 11), it can perform a circumvention to the left or to the right. However, the right circumvention is more efficient since it requires a smaller deviation from the original heading. Therefore, we project dashed lines that are parallel to the original flows and tangent to the CWC in order to determine the optimal circumvention path. Values ${X}_{dev}$ and ${Y}_{dev}$ present minimum lateral displacements of the flows ${F}_{1}$ and ${F}_{2}$ for which the aircraft will pass the CWC safely when the conflict resolution maneuvers are not considered.

### New IPt of the flows and the CZ projected flows tuning

**Fig. 9:**
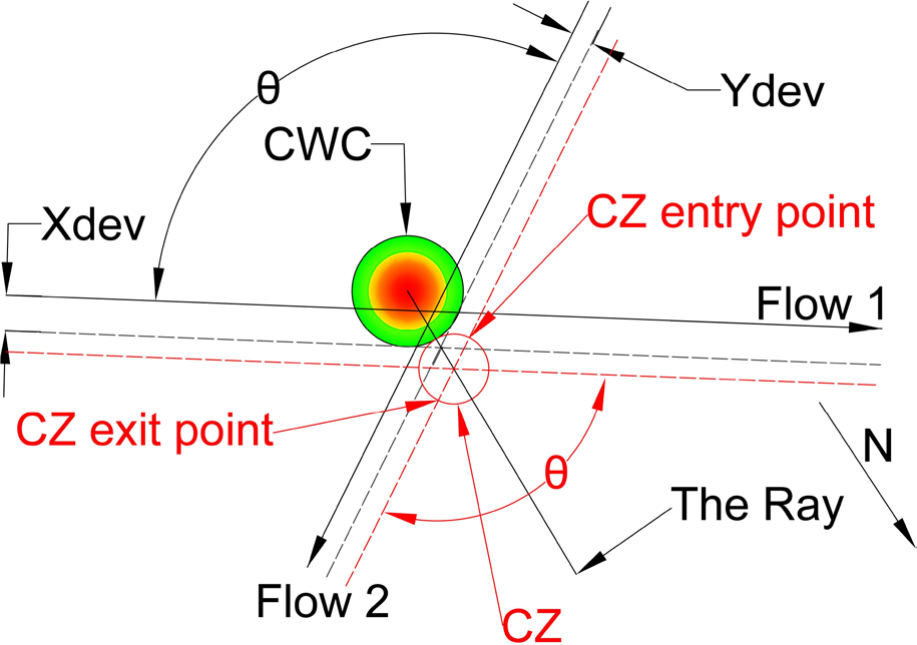
Projected ray and the CZ (red circle) for obtuse angle θ. Red dashed lines denote tuned projected flows of the CZ for intersection angle θ. The N denotes the direction of the North

Next we need to determine the new location of the IPt which satisfies that aircraft from both flows will not penetrate the area of the CWC. New IPt will be also the center of the CZ, so we need to provide that no overlapping between the CZ and the CWC area occurs. Therefore, the solution where the CZ is circle tangent to the CWC will satisfy these conditions, and hence all the CRM will be performed in the nearest safe distance from the CWC. The problem of positioning the CZ is solved by projecting the ray that emanates from the CWC center and passes through the IPt of dashed lines, and by positioning the CZ on the ray. By doing this, for any CZ radius, the CZ can be a circle tangent to the CWC. Also, the center of CZ will be at the same distance from both black dashed lines (original flows displacement), as long as it stays on the ray. The concept of projected ray and CZ tangent to the CWC is illustrated in Fig. 9.

**Fig. 10:**
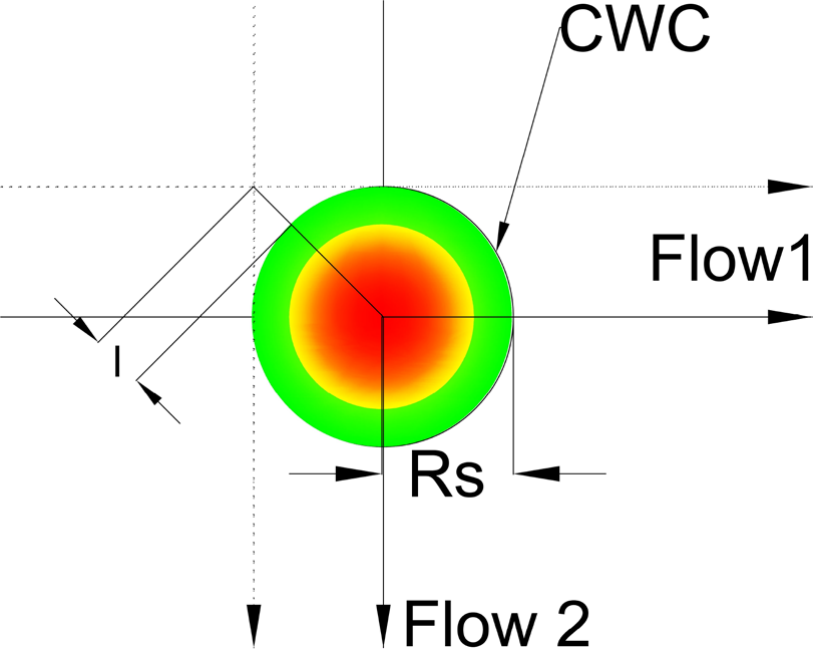
Flows intersecting at the 90° and the CWC centered at the IPt. For B = 1 and θ = 90° the CZ radius is *r* = 23.1066 NM

The IPt of two dashed lines (black) could be a new location of the IPt, thus it will provide minimum lateral displacement for the CWC during circumvention. However, for a given angle between dashed lines and the CWC radius, the CZ may overlap and penetrate the CWC. We can gain a better understanding of this by examining a symmetric example where the CWC is centered at the IPt of the original flows (Fig. 10).

The distance from the CWC center to the IPt of dashed lines is equal to:

6 $$s={R}_{S}\sqrt{2}=l+{R}_{S}=>l={R}_{S}(\sqrt{2}-1)$$

**Fig. 11:**
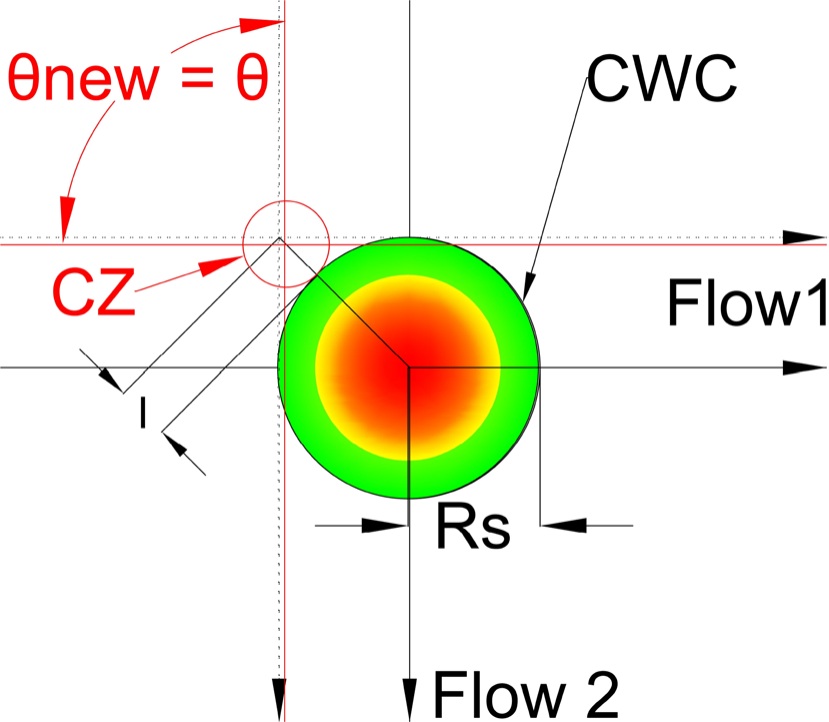
CZ moved closer to the CWC. In this case the CZ is centered closer to the CWC, so the projected flows are intersecting the area of the cell

In order to match the IPt of dashed lines with the CZ center, value *l* needs to be equal to *r*, and as CZ radius is *r* = 23.1066 NM (for B = 1 and θ = 90°) the CWC radius will be ${R}_{S}$ = 55.7843 NM. So, for the CWC radius ${R}_{S}$ = 55.7843 NM the CZ can be centered at the IPt of dashed lines, but if ${R}_{S}$ > 55.7843 NM then $l>r$. If such case happens, the one may think the CZ may be moved closer to the CWC, but if we do so the projected flows of the calculated CZ will intersect the CWC as it is illustrated in Fig. 11.

**Fig. 12:**
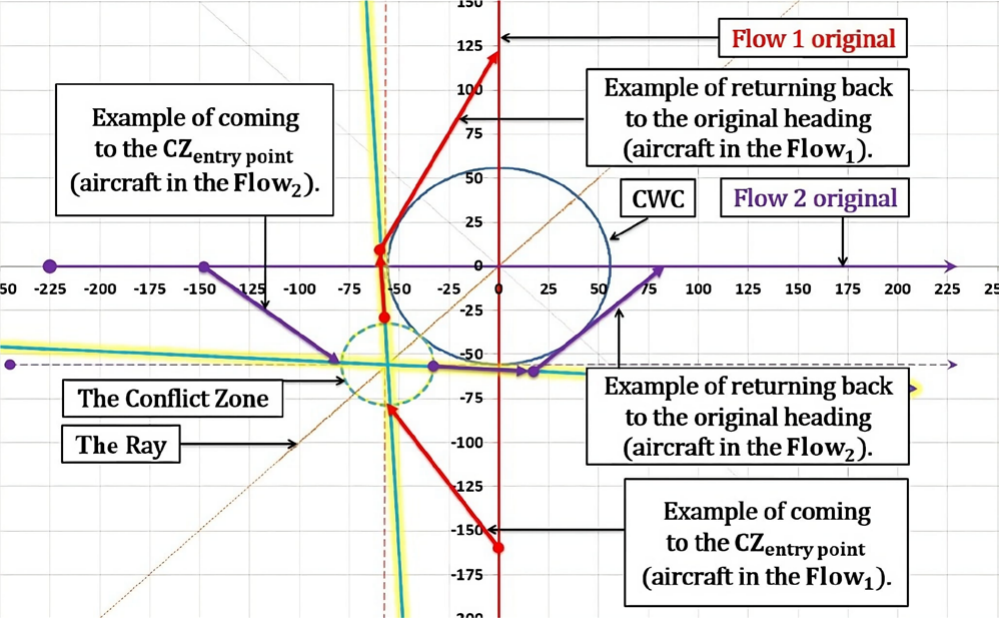
Flow 1 (red) is northbound, Flow 2 (purple) is eastbound, and the CWC is colored blue. The CZ is depicted as a dashed circle (light-blue with yellow shade) and projected new flows for the optimal angle of intersection are colored as the CZ

So, if we move the CZ closer to the CWC, we need to increase ${\theta }_{new}$ for a value that will provide no penetration through the CWC. An increase of the ${\theta }_{new}$ will cause change to the CZ radius (in this case CZ radius will decrease because it is closer to the ${\theta }^{*}$). Therefore, in this particular case, the solution will be the smallest possible CZ. As ${\theta }^{*}$ is 96° (for B = 1), projected flows will not intersect the CWC and at the same time the CZ will be the smallest possible, which is our goal. The solution for this particular case is illustrated in Fig. 12 (it is the same case viewed from different perspective) and it is obtained by analyzing the problem in the MicrosoftExcel2010.

If ${R}_{S}<55.7843 NM$ then $l<r$. The CZ center will need to be moved further from the CWC, so the CZ can become its tangent. Next, if projected flows intersect the CZ,${\theta }_{new}$ needs to be increased. We will try with angle ${{\theta }_{new}=\theta }^{*}$, and if projected flows still penetrate the CWC area, the ${\theta }_{new}$ would need to be increased until the flows are out of the CWC area.

After the ray projection, we need to examine possibilities for various new-projected flows angles of intersection. First, we will try with the ${\theta }^{*}$ as it provides the smallest possible CZ. So, we need to find a way to tune the new-projected flows for the ${\theta }^{*}$, and in the future for each CZ size we want to examine. Our goal is to set the flows equally from both sides of the CWC and that no penetration occurs with the CWC.

**Fig. 13:**
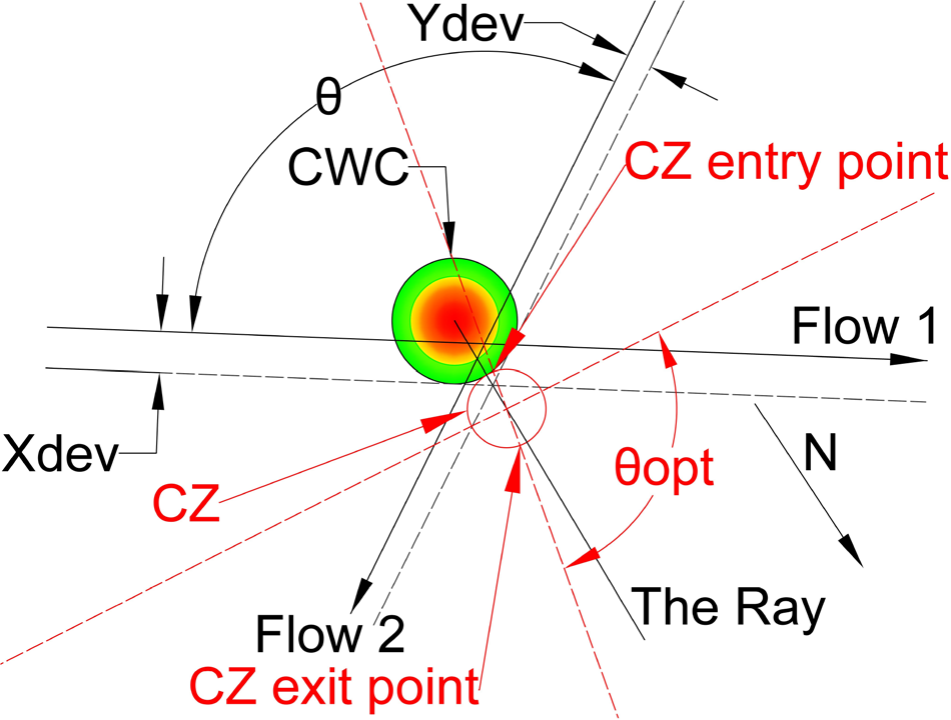
CZ projected flows tunning problem for the ${{\varvec{\theta}}}_{{\varvec{n}}{\varvec{e}}{\varvec{w}}\boldsymbol{ }{\varvec{o}}{\varvec{p}}{\varvec{t}}{\varvec{i}}{\varvec{m}}{\varvec{a}}{\varvec{l}}}$

In Fig. 13, the CZ with the projected flows is depicted; those flows are not tuned, so the new-projected Flow 2 passes through the CWC.

**Fig. 14:**
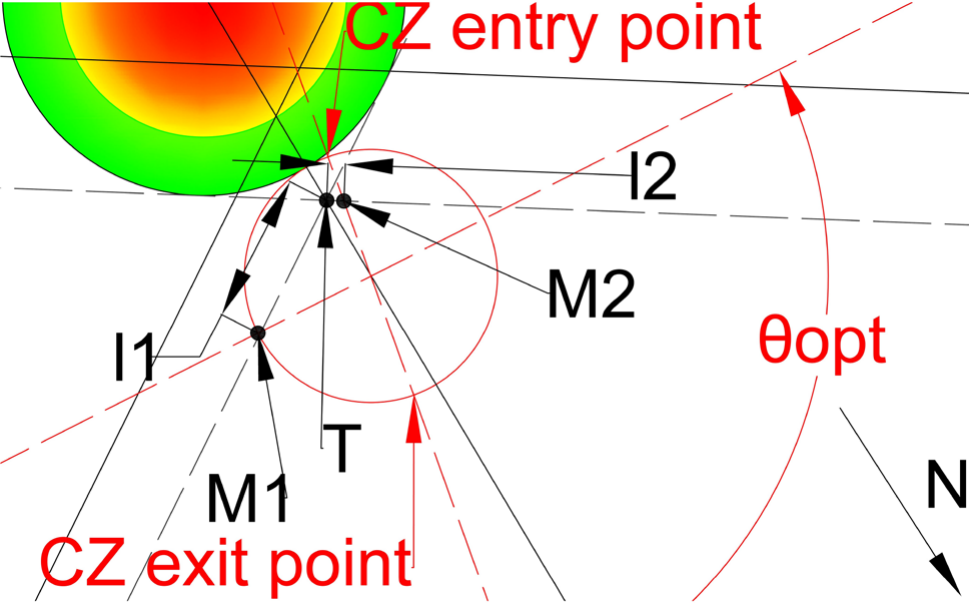
CZ projected flows tunning problem for the ${{\varvec{\theta}}}_{{\varvec{n}}{\varvec{e}}{\varvec{w}}\boldsymbol{ }{\varvec{o}}{\varvec{p}}{\varvec{t}}{\varvec{i}}{\varvec{m}}{\varvec{a}}{\varvec{l}}}$

**Fig. 15:**
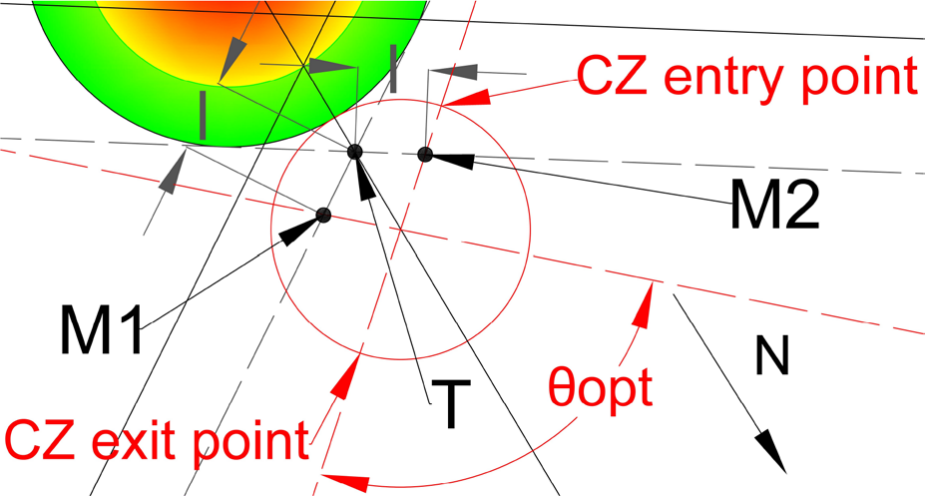
Tuned projected flows of the CZ

In order to find the solution for tuning, we will focus on the intersection point of dashed lines and the ray. Figure 14 depicts the scenario presented in Fig. 13; with the only variation being the lengths ${{\varvec{l}}}_{1}$ and ${{\varvec{l}}}_{2}$ used for the flows tuning. ${{\varvec{l}}}_{1}$ is the length from point **T** to point ${{\varvec{M}}}_{1}$, while ${{\varvec{l}}}_{2}$ is the length from point **T** to point ${{\varvec{M}}}_{2}$. We chose the point **T** to calculate the lengths ${{\varvec{l}}}_{1}$ and ${{\varvec{l}}}_{2}$, because the ray passes through that point and all the points on the ray are at the same distance from both projected flows of the CZ (red-dashed lines). ${{\varvec{M}}}_{1}$ and ${{\varvec{M}}}_{2}$ are points where the red-dashed lines intersect with the black-dashed lines for the flows ${F}_{1}$ and ${F}_{2}$ respectively. If we equalize lengths ${{\varvec{l}}}_{1}$ and ${{\varvec{l}}}_{2}$ (Fig. 15), the projected flows of the CZ will be at the same distance from the ray and thus from the CWC. By doing so we provide that neither one of the flows will have advantage from a distance in travel aspect.

**Fig. 16:**
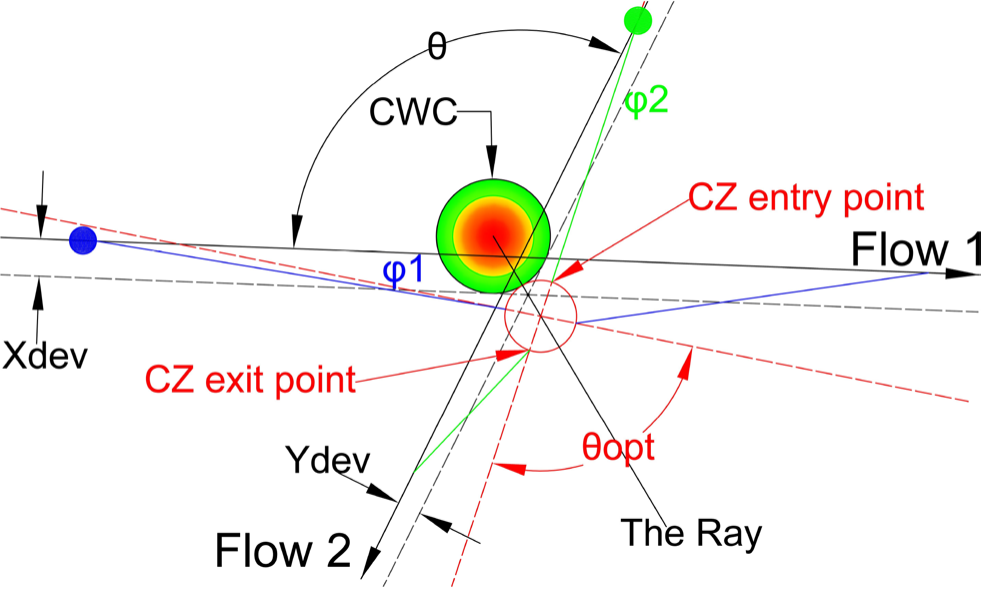
Conflict resolution in the case of the CWC circumvention with an obtuse angle of intersection between the flows

**Fig. 17:**
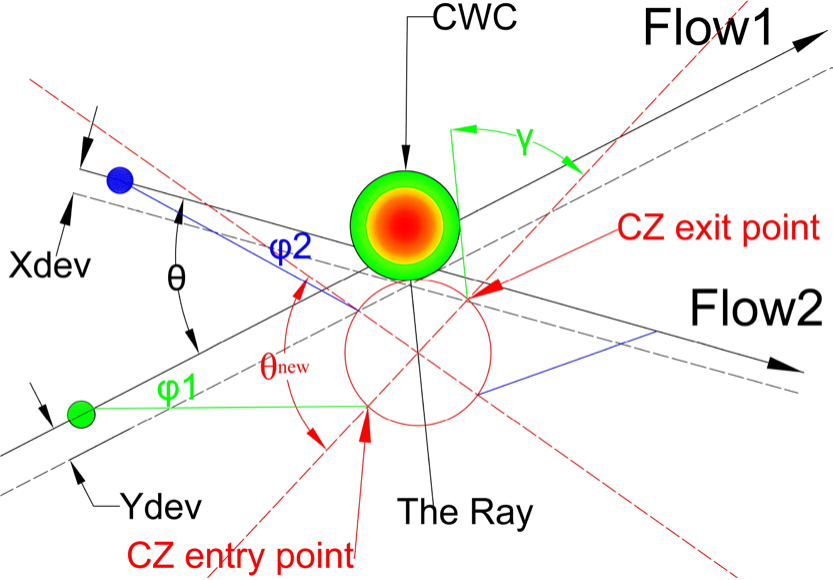
Conflict resolution in the case of the CWC circumvention with an acute angle of intersection between the flows

The final figure where both the conflict resolution and the CWC circumvention are provided is illustrated in Fig. 16. The aircraft in Flow 2 (green) adjusts its heading by a value of ${\varphi }_{2}$ to reach the CZ entry point. Once the conflict is resolved, the aircraft flies to the CZ exit point from which it can return to its original heading. The return to the original heading may involve taking the shortest possible path, it may depend on a next waypoint on the route, or it may be constrained by the CWC, if the circumvention has not been completed (Fig. 17). In the case illustrated in Fig. 16, both aircraft have already bypassed the CWC during the conflict resolution. As a result, returning to the original heading is noted by circumventing the CWC. Instead, they can proceed directly to their original tracks. In Fig. 17, for an acute θ example there is a slightly different situation. After the conflict resolution, the aircraft in Flow 1 has not completed the CWC circumvention, so in order to return to the original heading, its left turn is limited by the maximum heading deviation γ, which is preventing penetration through the CWC area.

## Analysis of the solution—a numerical example

We used the concept described in the previous section, as well as the MicrosoftExcel2010, to create the model for projecting relocated flows around the CWC. In order to get better insight in flows changing, we need to specify the CWC radius which is always centered at the origin of the X and Y axes. Additionally, we need to define the distances from the origin to the intersection points between Flow 1 and the X-axis, as well as between Flow 2 and the Y-axis.

The assumption of considering the CWC as a circle has simplified the building of the model, so the original Flow 2 is always a horizontal line parallel to the X-axis, and depending on the angle of intersection between the flows, Flow 1 is rotated. Next, we need to choose the angle of intersection between the original flows (θ) on the interval from 0° to 180°. After specifying the angle of intersection the model projects:

1. Dashed lines tangent to the CWC for both flows,
2. The ray that passes through the origin and the intersection point of the lines tangent to the CWC,
3. The optimal CZ (for the ${\theta }_{opt}$), that is centered on the ray and is the circle tangent to the CWC, and
4. Flows for the optimal CZ.

Besides abovementioned, the model generates new-projected flows centered on the ray with corresponding CZ $\left({CZ}_{new}\right)$. Also, it enables changing the values of the angle ${\theta }_{new}$ between those flows, which is useful for examining various sizes of the ${CZ}_{new}$ and comparing it to the optimal CZ.

**Fig. 18:**
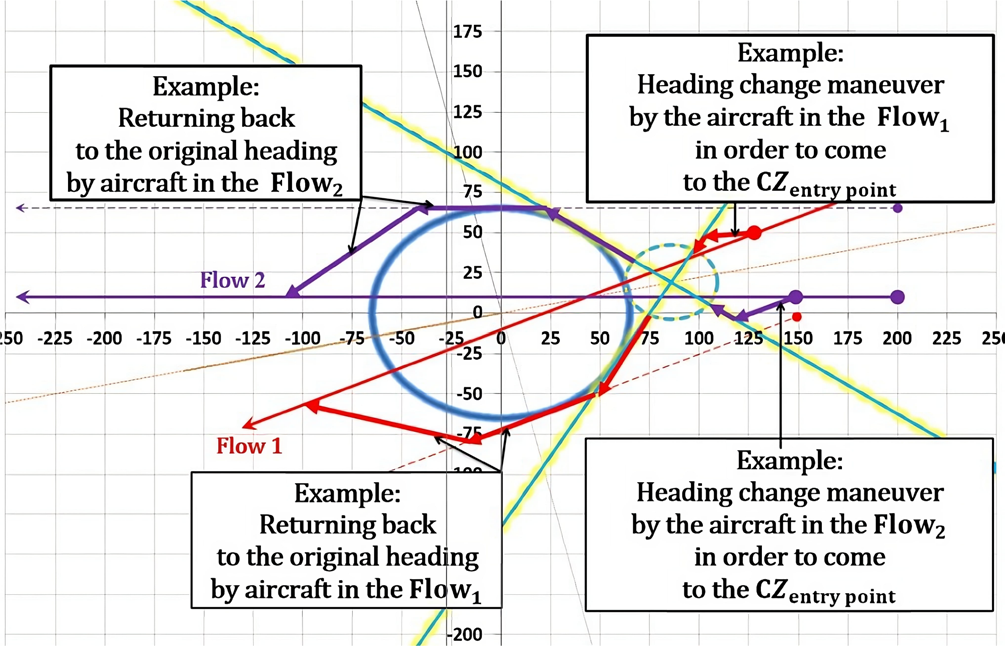
Case I: Conflict resolution and the CWC circumvention

**Fig. 19:**
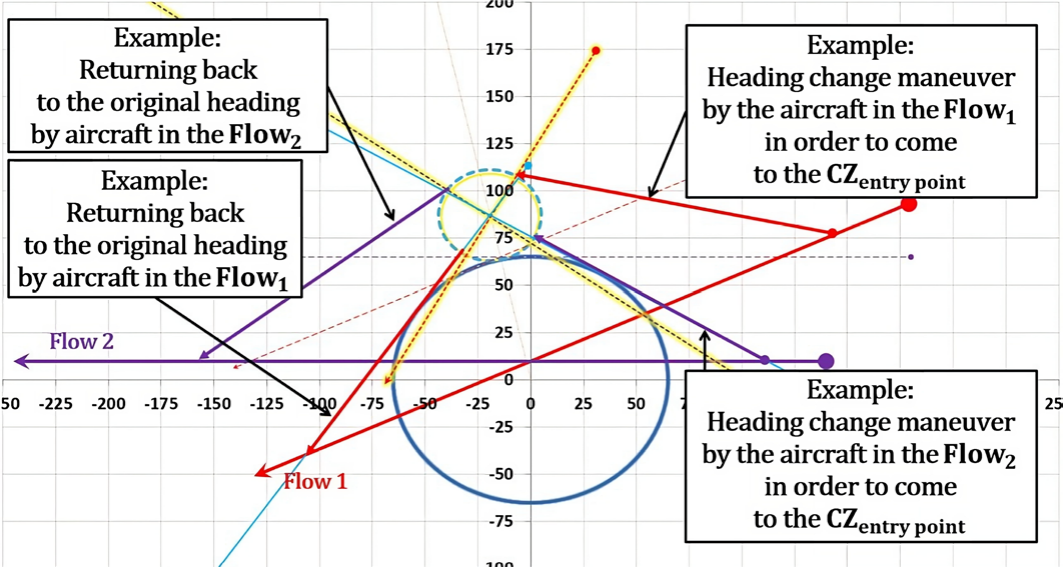
Case II: Conflict resolution and the CWC circumvention

**Fig. 20:**
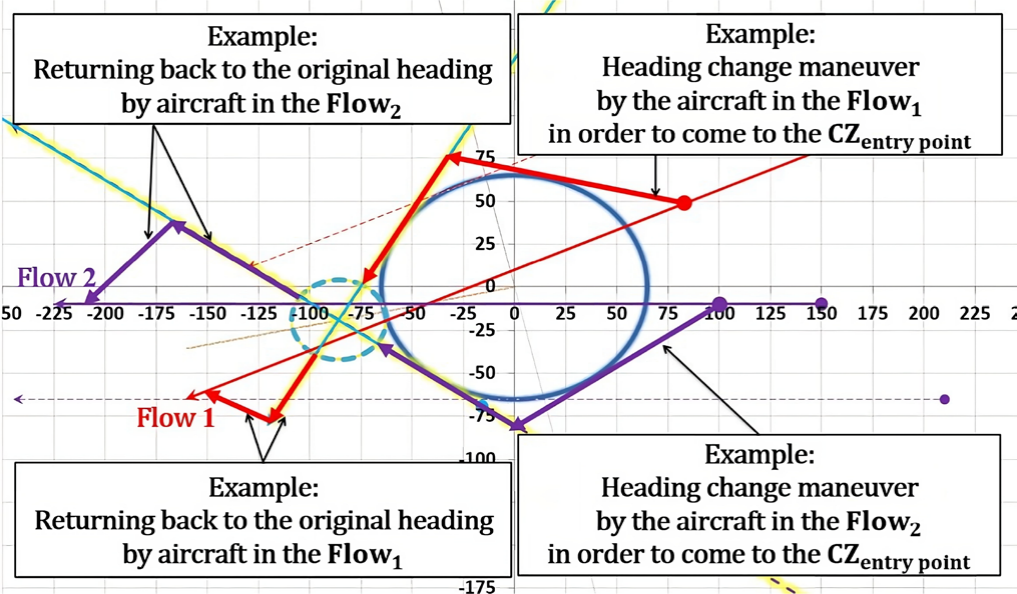
Case III: Conflict resolution and the CWC circumvention

The model presents a solution as a 2D diagram of projected flows (Figs. 18, 19, 20, etc.). Therefore, depending on the original flows intersection with X and Y axes, the CZ is always in one of four quadrants or in between them. For example, if the original flow 1 intersects the positive X-axis, then the line tangent to the CWC will also intersect the positive X-axis. By doing so, we provide that aircraft performs circumvention of the CWC from a side where the circumvention requires less deviation from the original flow. In general, there are four cases for all angles of intersection. Herein will be presented all four of them for an example where: the angle between the original flows is θ = 25°, the angle between the new-projected flows is the optimal one (${\theta }_{new}$ = 96°), the radius of the CWC is ${R}_{S}$ = 45NM, and for buffer coefficient B = 1.

### The first case

In the beginning both aircraft in the original flows fly to the CZ to perform the CRMs, and upon resolving potential conflict they continue to or initiate the CWC circumvention (Fig. 18). In this configuration, the CZ center and the CWC center are always positioned between inner sides of both original and new-projected flows. In other words, after the intersection point, the CWC is positioned to the right of the aircraft in the new flow 1 and to the left of the aircraft in the new flow 2. By projecting the flows in this manner, greater radii of the CWC can be more easily circumvented. This is because the CWC is positioned on the wider side of the flows' intersection, and the circumvention phase is not constrained by the conflict resolution phase. As a result, more space is available for the aircraft to maneuver and avoid the CWC, leading to a more efficient and effective solution. If it happens that new-projected flows for ${\theta }_{opt}$ penetrate the CWC, the angle of intersection will need to be increased until the flows are at least lines tangent to the cell. In this example that will be the case if the CWC radius is greater than 46.6NM (66.6NM accounting the minimum safety buffer).

### The second case

In such configuration, aircraft in the new-projected flow 1 first resolves potential conflict with an aircraft in the new-projected flow 2, while just initiating the CWC circumvention. After the conflict resolution this aircraft needs to circumvent much greater portion of the CWC compared to the flight prior the conflict resolution. The opposite case is with the aircraft in the new-projected flow 2, which circumvents the greater portion of the CWC before coming to the CZ. The CWC is positioned on the left side of the new-flows, which is not the wider side of the intersection as it was in the previous case, hence ${\theta }_{opt}$ is not feasible in this case. Consequently, angle ${\theta }_{new}$ must be decreased in order to prevent penetration of the aircraft through the cell, which entails increase of the CZ area. Therefore, as far as the space utilization is considered, this case illustrated in Fig. 19 is inferior comparing to the 1st case.

### The third case

Sequences in this configuration are opposite of those described in the 1st case. Prior the conflict resolution both aircraft complete the CWC circumvention and then proceed to the CZ (Fig. 20). Again, the CZ and CWC centers are positioned between the inner sides of the original and new-projected flows, which allow circumvention for greater radii of the CWC. In this case, comparing to the 1st case, returning to the original heading is shorter because of the opposite sequences of conflict resolution and CWC circumvention, but it is neutralized due to initial deviation from the original heading (circumvention manuevers). From the space utilization aspect, this configuration is equally efficient as the first one, and is superior comparing to the other two.

### The fourth case

**Fig. 21:**
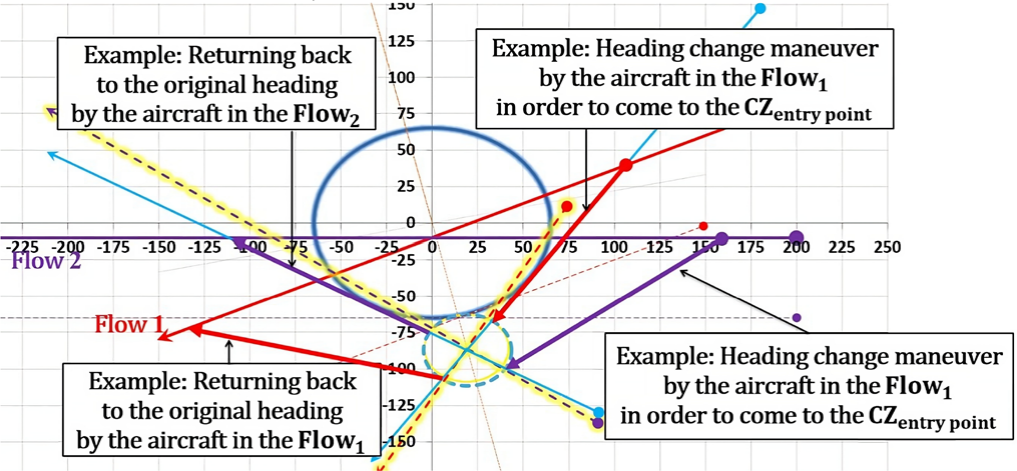
Case IV: Conflict resolution and the CWC circumvention

The fourth case, illustrated in Fig. 21, is similar and equally efficient as the 2nd one. Here the aircraft in the new-flow 1 circumvents the greater portion of the CWC prior the conflict resolution and completes it after the conflict resolution, while aircraft in the new-flow 2 does the opposite thing. As in the 2nd case, the CWC is positioned on the side of the new-flows which is not the wider one, so ${\theta }_{new}$ needs to be smaller than the ${\theta }_{opt}$ in order to provide circumvention. The highest possible value that can be applied here is 86°, (CZ radius of 24.247 NM).

The examples illustrated above are cases for acute angle of intersection between the flows, but nevertheless, four cases of intersection are valid for obtuse angles as well.

We saw that 1st and 3rd cases are more efficient than the other two. This is due to configuring the flows intersection in a way that provides positioning the CWC right between the flows. Also, from other two examples where the ${\theta }_{opt}$ could not be applied, we saw the principles of finding the most efficient angle (from the aspect of the CZ size) that provides safe circumvention.

**Fig. 22:**
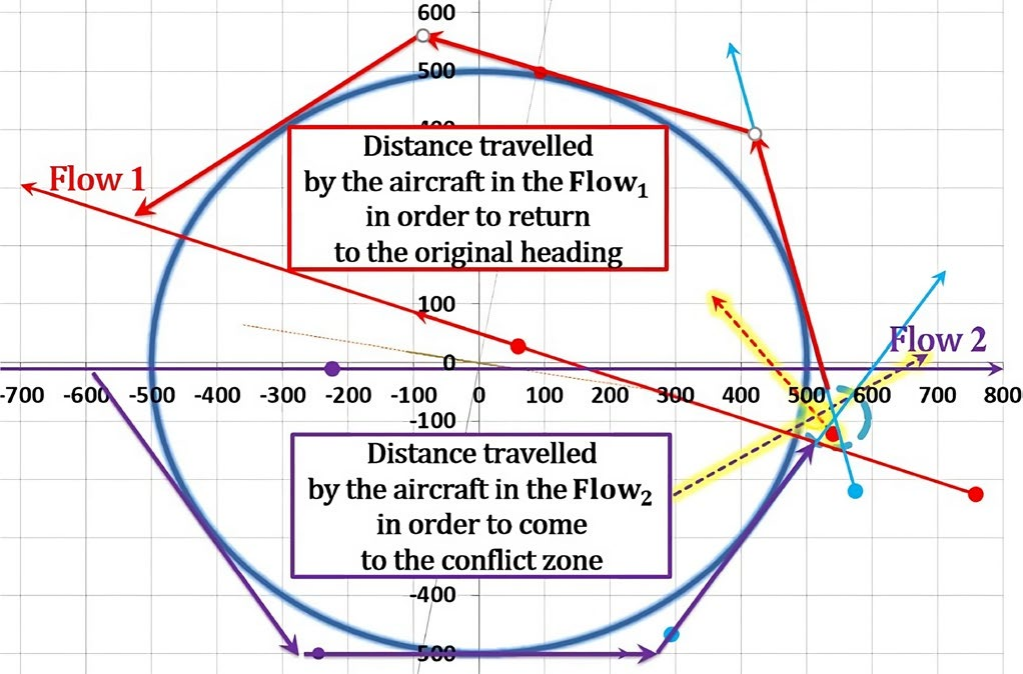
Circumvention and the conflict resolution of the CWC of a radius ${{\varvec{R}}}_{{\varvec{S}}}=500{\varvec{N}}{\varvec{m}}$. In this example, aircraft in the Flow 1 first needs to come to the CZ then to circumvent the CWC, while aircraft in the Flow 2 needs to do the opposite

As it can be expected, desirable angles of intersection between flows are those around the value of the optimal one. In some cases, those angles could not be applied to the problem (2nd and 4th cases) hence other values need to be considered. We saw in the 2nd and 4th case that ${\theta }_{opt}$ does not provide the solution; hence the intersection angle between the new-projected flows had to be decreased. This can lead to thought that in cases of extremely large CWC the ${\theta }_{new}$ will need to be very small, which implies in inefficiency of the conflict resolution for small angles (Fig. 5). Therefore, we simulated the case of an enormously large CWC, such as one of the radius ${R}_{S}=500 NM$ (Fig. 22). In reality, this is not the case, and even if it could happen, probably all flights that need to pass through that region would be cancelled due to fuel and time inefficiency caused by the circumvention needs. Thus, we simulated this case only to demonstrate the ability of the model to find a solution even in extreme and hardly imaginable cases. The MicrosoftExcel2010 model found that the new-projected flows can provide safe circumvention with the angle of 48° between them (${\mathrm{r}}_{\mathrm{CZ}}=51.163 NM$). As mentioned before, aircraft would need to travel huge distances in order to come to the CZ, leave it and return to the original flows. Such case is illustrated in Fig. 22.

The problem solved in this article could be explored more thoroughly if large and complex sets of data related to weather patterns, aircraft performance, and flight routes (i.e. real-time convective weather cell data, aircraft position and altitude, etc.) are taken in consideration. This data could be sourced from weather sensors, flight tracking systems, and aircraft telemetry. Applying advanced analytical techniques to the complex data sets, such as machine learning algorithms or data mining, could lead to improved results, providing more insights to the factors that affect air traffic management. For example, analysis of convective weather cell and flight data, could enable air traffic controllers make more informed decisions, and potentially improve safety and efficiency of air traffic management.

If algorithms need acceleration, due to Big Data, possibilities are: (a) The existing computing architecture could be enhanced [24], (b) A new computing architecture could be introduced [25], (c) The number of iterations of the algorithm could be minimized using machine intelligence [26], or (d) Each iteration could be cut shorter if some kind of suboptimal computing is utilized [27].

## Extension to other field of study

After discussing the main focus of the research, it is important tohighlight its potential contribution to other field of study.

At the first glance, assumptions mentioned in the section Proposed Solution, such as the CWC to be static and of circular shape, are not describing the real-world scenarios related to the CWC behavior. However, these assumptions become eligible and relevant if we take in consideration a conflict resolution between unmanned aerial vehicles (UAV) and fixed objects or any constrained area approximated with a circle that requires to be bypassed. In other words, the stated assumptions become relevant when considering conflict resolution between UAVs or drones instead of aircraft, as well as when examining the circumvention of a fixed object (such as building), or a constrained area where UAV flights are prohibited (such as an airport/heliport flight restricted zone). Therefore, with slight modifications, the principles of the model could also be applied in this field of study.

## Conclusion

Building upon the work conducted by Mao et al. related to conflict resolution and flows intersection, we extended the problem by incorporating a constrained area, such as the CWC, and took into consideration its simplified form. We proposed a solution for conflict avoidance between aircraft, as well as between aircraft and the CWC, when aircraft are required to bypass the restricted area. The proposed solution has been tested and analyzed in the MicrosoftExcel2010 model, and the analysis demonstrated that depending of the size of the CWC and the buffer coefficient, solutions to the CWC circumvention and the conflict resolution could always be found. In cases where the CWC is extremely large, a solution can be achieved at the cost of increased distances in circumvention due to huge deviations between the original flows and the new-projected flows. The analysis also highlighted the significance of configuring the intersection of flows around the CWC. Specifically, when the CWC is positioned between inner sides of the new-projected flows (where both aircraft either resolve conflict or initiate CWC circumvention first), the flows can intersect at angles that require smaller CZs areas. This is in contrast to other cases where the CWC is positioned on the outer sides of the new-projected flows.

The proposed solution addresses problem of conflict resolution between two aircraft inclose proximity to the CWC, and provides insights into the size of occupied airspace required for the conflict resolution. This can be beneficial to ATCo, particularly when high intensity of upcoming air traffic is expected and when sectors capacities are reduced due to severe weather conditions.

This article focuses on reducing the airspace required for aircraft-to-aircraft and aircraft-to-weather conflict resolution. It differs from current best solutions and industry practices, which primarily emphasize distance travelled, time savings, and fuel consumption minimization.

Exploring the problem more thoroughly might include consideration of complex data sets related to weather and flight data. We believe that conducting more sophisticated analyses on such data sets, and taking advantage of Big Data, could lead to improved results and contribute to overall safety and efficiency of the air traffic management. Thus this article could provide good foundation and huge potential for future research related to this field of study.

Developing an automated tool for finding the smallest possible areas for the conflict resolution, especially in cases of the CWC impact, could provide better utilization of the airspace, and hence help in recuperating the reduced capacity of the affected sectors.

Future work could be focused on examining the conflict resolution in the cases when the CWC is moving. This would be the step further in finding the solution that could be applied to the real-world air traffic problems. Additionally, approximation of CWCs with irregular shapes would enhance the research and provide deeper understanding of the problem. Future work may take into account multiple flows intersecting within the CWC area, thus taking us another step closer to the real-world scenarios. In that case, the computational aspect may become complex, but this challenge could be overcome by the utilization of a more proper computing paradigm (e.g., [29]).

As the phenomenon and usage of the unmanned aerial vehicles becomes more popular, the need for resolving potential conflicts in UAVs field, whether between themselves or with other objects or constrained areas, will continue to grow. In this context, the proposed solution can provide valuable insights into the airspace required for effective resolution of conflicts [30].

## Data Availability

Datasets could be provided upon request from authors.
